# Gut microbiota-ferroptosis signaling as a putative feed-forward amplifier of pulmonary immunometabolic reprogramming in acute respiratory distress syndrome: actionable therapeutic targets

**DOI:** 10.3389/fimmu.2026.1900118

**Published:** 2026-10-28

**Authors:** Biying Dong, Weitong Zeng, Bing Zhong, Longxiong Liao, Yi Wei, Lihua Liao, Dongwei Zhang, Xianming Fan

**Affiliations:** 1 Department of Respiratory and Critical Care Medicine, Liuzhou People’s Hospital, Guangxi Medical University, Liuzhou, China; 2 Key Laboratory of Diagnosis, Treatment and Research of Asthma and Chronic Obstructive Pulmonary Disease, Liuzhou People’s Hospital, Guangxi Medical University, Liuzhou, China; 3 Department of Respiratory and Critical Care Medicine, The Affiliated Hospital of Southwest Medical University, Luzhou, China; 4 Inflammation & Allergic Diseases Research Unit, The Affiliated Hospital of Southwest Medical University, Luzhou, China; 5 Fever Clinic, Liuzhou People’s Hospital, Guangxi Medical University, Liuzhou, China

**Keywords:** acute respiratory distress syndrome, ferroptosis, gut microbiota, gut–lung axis, inflammation, macrophage polarization, metabolic reprogramming

## Abstract

Acute respiratory distress syndrome (ARDS) is characterized by dysregulated pulmonary immune responses, and effective targeted therapeutic strategies remain an unmet clinical need. Accumulating preclinical and clinical correlative data reveal complex interconnections between gut microbiota and ferroptosis-driven immunopathology in ARDS, yet an integrated mechanistic understanding of the gut-lung pathological axis remains incomplete. This review puts forward a testable conceptual temporal framework for ARDS-related gut-lung crosstalk. Evidence supporting the gut-to-lung limb is comparatively more abundant, whereas the full sequential pathogenic cascade has not been rigorously validated. Gut-derived metabolites remodel pulmonary cell phenotypes, macrophage functional states, neutrophil extracellular trap formation and T-cell homeostasis, potentially sustaining a self-amplifying ferroptosis-inflammatory vicious cycle that may drive pulmonary immunometabolic reprogramming. Targeting this regulatory cascade may yield promising conceptual therapeutic avenues, including microbiota-modulating interventions, anti-ferroptotic strategies and multi-omics-guided precision approaches. Major knowledge gaps persist: most mechanistic observations are extrapolated from non-ARDS animal models; temporally-separated preclinical experiments that induce lung injury prior to gut-targeted manipulation are scarce; and well-controlled human studies disentangling pulmonary-driven effects from ICU confounders are urgently required. This sequence represents an etiology-dependent working model rather than a uniform chronological hierarchy for all ARDS. In direct-pulmonary ARDS, alveolar injury may precede gut dysbiosis; in indirect/sepsis-associated ARDS, systemic inflammation, intestinal hypoperfusion and antibiotic exposure may occur before, or simultaneously with, overt pulmonary ferroptotic injury.

## 1 Introduction: ARDS as an immune-driven disorder

This review proposes a conceptual, evidence-tiered framework centered on the gut–lung axis rather than asserting a proven causal cascade (1). We hypothesize that ARDS-related insults (e.g., sepsis, infection, direct inflammatory triggers) may first elicit early alveolar ferroptotic injury (Phase 1a) (2). Through systemic inflammation, splanchnic hypoperfusion and hypoxia, this pulmonary injury may subsequently contribute to intestinal barrier disruption and gut dysbiosis (Phase 1b) (1). However, this pulmonary-to-gut directional sequence has not been directly proven in ARDS human cohorts or dedicated preclinical models (1). This is only one possible trajectory (proposed, etiology-dependent), not a general ARDS pathogenic model. In indirect/sepsis-associated ARDS, intestinal barrier dysfunction and gut dysbiosis can arise prior to or concurrently with pulmonary injury. Once gut dysbiosis is established under this hypothetical scenario, gut-derived metabolites may feed back into the pulmonary injury loop through the gut–lung axis (3). Circulating gut-derived metabolites—such as SCFAs (4) and other microbial byproducts—may translocate systemically and modulate pulmonary ferroptosis, macrophage polarization (5) and efferocytosis, and downstream inflammatory responses, potentially perpetuating lung injury. Dickson et al. provided the landmark observation of lung microbiome enrichment with gut bacteria in sepsis-associated ARDS, establishing the foundational evidence for gut-to-lung microbial translocation (6). This direction has been subsequently confirmed and extended by a 2025 systematic review incorporating 11 studies and 660 patients, which corroborated the reproducible enrichment of gut-associated Enterobacteriaceae in ARDS lungs and its association with fewer ventilator-free days and mortality (7). Longitudinal multi-compartment analyses further demonstrated dynamic host-microbiota interactions across pulmonary, intestinal, and systemic compartments in acute respiratory failure (8). Additional 2024 cohort studies have characterized lung microbiota composition across ARDS etiologies, demonstrating that microbiota signatures differ between influenza, COVID-19 and bacterial ARDS (9, 10). However, it should be emphasized that none of these studies directly establish the reverse pulmonary-to-gut causal direction; this study should not be cited as direct evidence for that sequence.

The Phase 1a-1b-2 sequential cascade represents a testable working hypothesis rather than an experimentally validated pathogenic sequence (see **Box 1** for full definition of Phase 1a-1b-2 working model).

Box 1 **Definition of the proposed Phase 1a-1b-2 etiology-dependent working model.**

Proposed Phase 1a-1b-2 represents an etiology-dependent working model rather than a universal chronological pathogenic hierarchy for all ARDS subtypes.

• Phase 1a: ARDS-associated pulmonary insult may initiate early alveolar ferroptotic injury.

• Phase 1b: Pulmonary-injury-derived systemic inflammation, splanchnic hypoperfusion and hypoxia may secondarily contribute to intestinal-barrier disruption and gut dysbiosis. The pulmonary-to-gut directional sequence remains unproven in dedicated human ARDS cohorts and ARDS animal models. This represents only one plausible trajectory and is not mandatory; in indirect/sepsis-associated ARDS, intestinal barrier dysfunction and gut dysbiosis can occur prior to or concurrently with pulmonary injury.

• Phase 2: Once gut dysbiosis is established, translocated gut-derived microbial metabolites may serve as putative feed-forward amplifiers exacerbating pre-existing pulmonary ferroptosis and immunometabolic injury.

Evidence stratification:

1. The gut-to-lung limb (gut-derived signals influencing lung pathology): supported by multiple correlative clinical observations and pre-clinical sepsis-ALI experiments.

2. The pulmonary-to-gut limb (lung injury driving intestinal disturbance): mechanistically plausible but unvalidated for ARDS; heavily confounded by ICU clinical interventions.

3. The full Phase 1a-1b-2 sequential feed-forward cascade: a testable working hypothesis, not experimentally-validated pathogenic cascade.

It is critical to distinguish different levels of available supporting evidence for this bidirectional cascade. The gut-to-lung limb (metabolites/bacteria from gut influencing lung pathology) is supported by multiple correlative clinical observations and pre-clinical sepsis-ALI experiments (3, 11). In contrast, the pulmonary-to-gut limb (lung injury driving intestinal disturbance) rests largely on cross-sectional clinical associations and indirect extrapolations from general sepsis models (1). The complete sequential loop Phase 1a→1b→2 representing pulmonary injury → gut dysbiosis → metabolite-mediated pulmonary amplification still constitutes an open, testable hypothesis rather than an experimentally validated pathogenic sequence (1, 3). In brief, three tiers of evidence can be distinguished for this gut-lung axis framework: (1) The gut-to-lung limb: supported by sepsis-ALI pre-clinical experiments and correlative human clinical observations, yet direct causal evidence specific to human ARDS remains limited. (2) The pulmonary-to-gut limb: mechanistically plausible but mainly extrapolated from sepsis, hemorrhage and ischemia-reperfusion animal models, without dedicated ARDS-specific proof. ICU-related confounders complicate causal interpretation.

Another key experimental limitation concerns the temporal logic of Phase 1b → Phase 2. Most existing pre-clinical studies deliver microbial or metabolite-based interventions concurrently with pulmonary-insult modeling (12, 13). Few studies adopt sequential experimental designs: inducing pulmonary injury first, and only afterwards perturbing or repairing gut barrier and gut microbiota. As a result, direct experimental evidence demonstrating that secondary gut dysbiosis amplifies pre-existing pulmonary injury after ARDS onset remains incomplete, representing an important unresolved research gap in this field.

Our review assembles an integrative conceptual framework synthesizing existing published evidence that connects gut microbiota, microbial metabolites, pulmonary ferroptosis, and immune-remodeling processes. Previous reviews have separately covered gut-lung crosstalk or ferroptosis-mediated lung injury. Building on prior work, we present a stepwise exposition: Chapter 2 details ferroptosis-driven immune derangements in the lung; Chapter 3 covers ARDS-associated intestinal dysbiosis; Chapter 4 explores how gut-derived metabolites exert bidirectional modulation on ferroptosis; and Chapter 5 systematically categorizes therapeutic strategies targeting each stage of this regulatory cascade.

Acute respiratory distress syndrome (ARDS) is a life-threatening clinical disorder characterized by bilateral pulmonary infiltrates, refractory hypoxemia, and non-cardiogenic pulmonary edema, arising from diffuse alveolar damage and dysregulated inflammation (14, 15). Despite progress in understanding its complex pathophysiology, ARDS remains heterogeneous in etiology, phenotype, and treatment response, severely limiting effective clinical management (16, 17). Currently, care relies mainly on lung-protective ventilation, and no pharmacotherapies targeting core pathogenic mechanisms have been approved (18–21). The lack of validated biomarkers and precise subphenotyping strategies further restricts personalized treatment, leaving substantial gaps between preclinical findings and clinical translation. Bos & Ware provided the seminal framework for ARDS phenotypic heterogeneity (14), distinguishing hyperinflammatory and hypoinflammatory subphenotypes with divergent treatment responses. This framework has been substantially refined by recent longitudinal multi-omics analyses: integrative metabolomic and transcriptomic profiling of 160 ARDS patients from the ROSE trial identified four molecular signatures associated with mortality, revealing distinct pathways including enhanced innate immune activation, hepatic dysfunction, and redox impairment (22). Large-scale multi-centre proteomic profiling has further validated distinct inflammatory phenotypes with potential therapeutic implications (23). Comprehensive reviews of omics technologies in ARDS have consolidated these advances, establishing metabolomic biomarkers such as phenylalanine and choline for severity assessment and subphenotype stratification (24). The COVID-19 pandemic has starkly underscored these unmet needs (25).

Emerging evidence identifies ferroptosis, an iron-dependent, non-apoptotic cell death driven by lipid peroxidation, as a key driver of alveolar epithelial and endothelial damage in ARDS. Its core features — iron overload, GPX4 (glutathione peroxidase 4) inactivation, and reactive oxygen species accumulation — are intricately linked to the disease’s pathological cascade (26–28). It is worth noting that our team has previously summarized the general role of gut microbiota and its metabolites in ARDS (29). However, that earlier work did not systematically dissect the mechanistic crosstalk between gut microbiota and ferroptosis, nor did it establish an integrative conceptual regulatory framework linking metabolic reprogramming, immune microenvironment remodeling, and ferroptosis in ARDS. The present review extends beyond our previous work and assembles an integrative conceptual “gut microbiota–ferroptosis” regulatory framework, focusing on bidirectional signaling loops shaped by specific microbial metabolites and regulated cell-death pathways.

The gut microbiota remotely modulates pulmonary homeostasis via the gut-lung axis, with microbial metabolites (e.g., short-chain fatty acids, indole derivatives) shaping systemic immune and metabolic landscapes (30). Growing evidence reveals intensive bidirectional crosstalk between gut microbiota and ferroptosis across multiple systemic diseases (27, 31). Drawing upon these observations, we elucidate how gut-microbiota-ferroptosis crosstalk may amplify ARDS progression through dual roles in metabolic reprogramming and immune modulation, discuss translational considerations, and outline future directions for precision medicine. We first delineate how ferroptosis drives immune dysregulation in ARDS, then examine gut microbiota-immune crosstalk via the gut-lung axis, followed by molecular mechanisms through which microbial metabolites control ferroptosis in immune cells, and finally discuss therapeutic opportunities.

To systematically visualize the proposed gut-microbiota-metabolite-ferroptosis-immune loop and resolve fragmented descriptions of upstream and downstream signaling, we constructed an integrated schematic as **Figure 1**. Panel A provides the organ-level panoramic temporal framework of the gut-lung pathological cascade; Panel B summarizes evidence-strength stratification for bidirectional gut-lung signalling. **Figure 2** details the intracellular molecular and cellular mechanisms, while **Figure 3** summarizes translational therapeutic strategies annotated by evidence grade.

**Figure 1:**
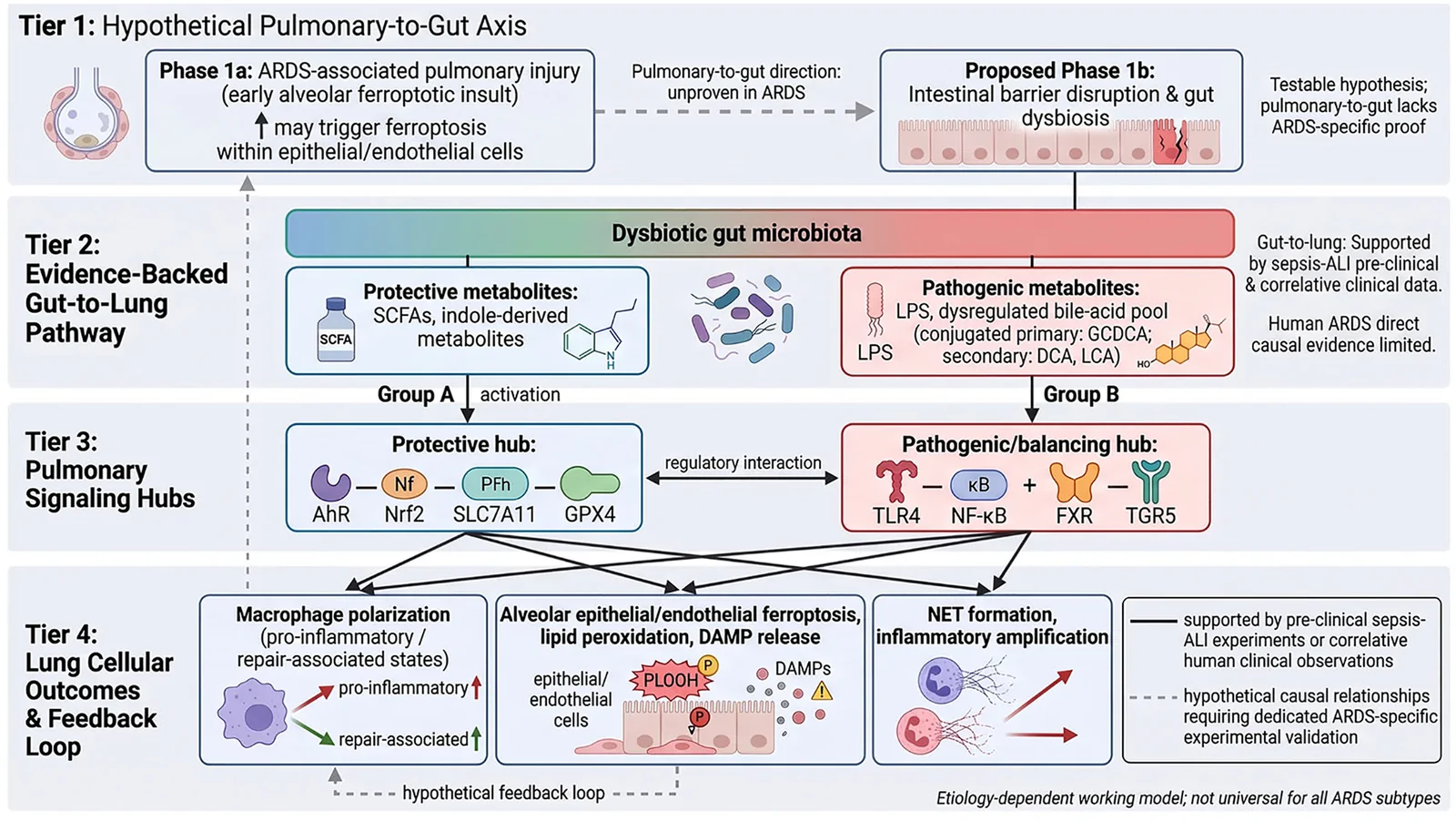
Tiered mechanistic framework for gut microbiota-ferroptosis signalling along the gut-lung axis in ARDS. Tier 1 illustrates the hypothetical pulmonary-to-gut limb of the Phase 1a-1b-2 cascade: ARDS-associated alveolar injury is proposed to disrupt intestinal barrier integrity and induce gut dysbiosis, but this directional causality lacks direct experimental validation in ARDS (see **Box 1** for full definition of Phase 1a-1b-2 working model). Tier 2 depicts two classes of circulating metabolites originating from dysbiotic gut microbiota, subdivided into protective and pathogenic metabolite pools. Tier 3 shows two core pulmonary signalling hubs that integrate microbial metabolite signals to regulate ferroptosis and inflammatory responses. Tier 4 summarises downstream alveolar cellular consequences, including macrophage functional states, epithelial/endothelial ferroptosis and NET formation, alongside a hypothetical feedback loop that may amplify initial pulmonary injury. Solid black lines indicate pathways supported by sepsis-ALI pre-clinical experiments or human correlative clinical observations. Dashed grey lines represent hypothetical causal relationships requiring dedicated ARDS-specific experimental validation. This etiology-dependent working model is not universally applicable to all ARDS subtypes.

**Figure 2:**
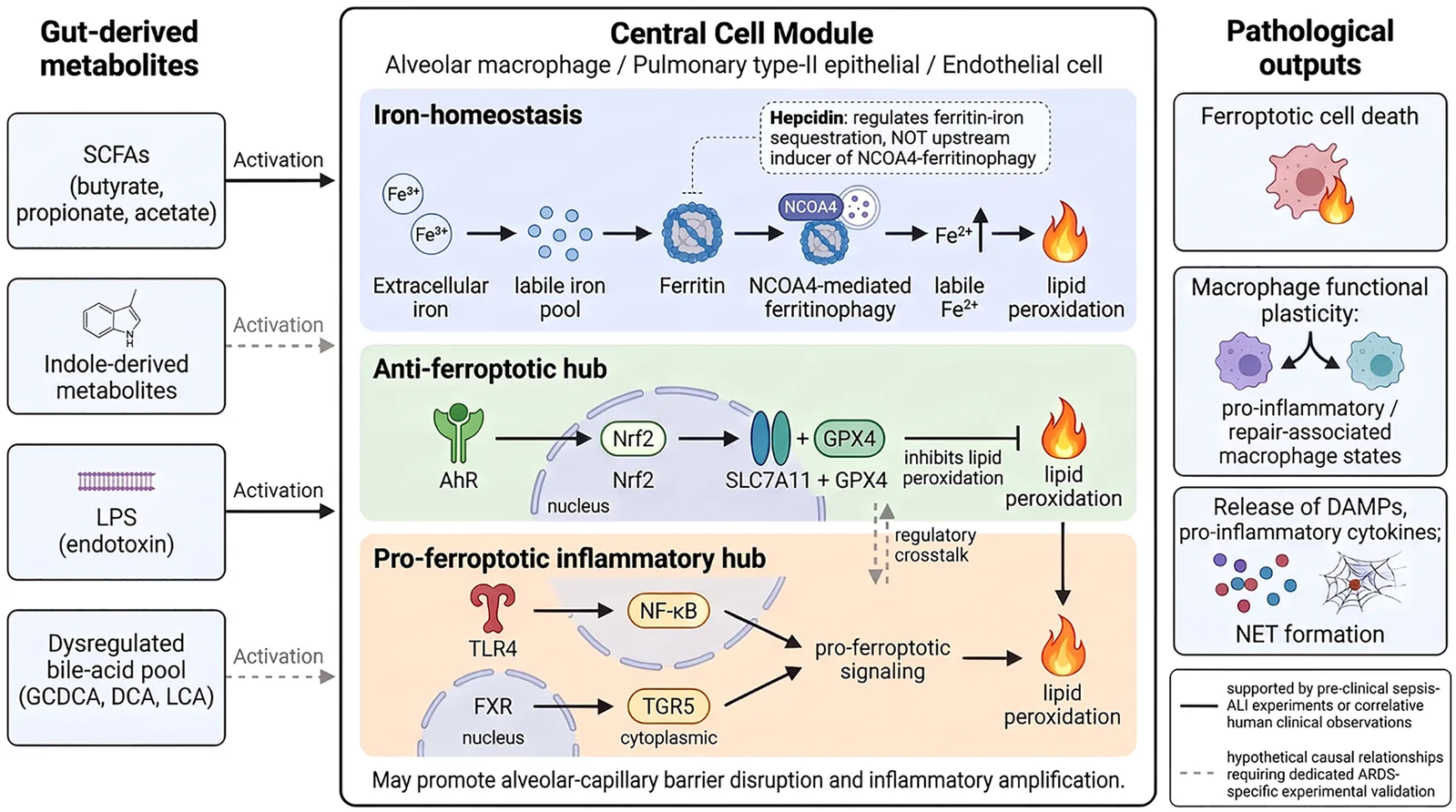
Mechanistic diagram illustrating gut-derived metabolites that modulate pulmonary ferroptosis during ARDS. Solid lines represent associations supported by pre-clinical sepsis-ALI experiments or correlative human clinical observations. Dashed lines denote hypothetical causal relationships that require dedicated ARDS-specific experimental validation. Hepcidin regulates ferritin iron sequestration and is not an upstream inducer of NCOA4-mediated ferritinophagy.

**Figure 3:**
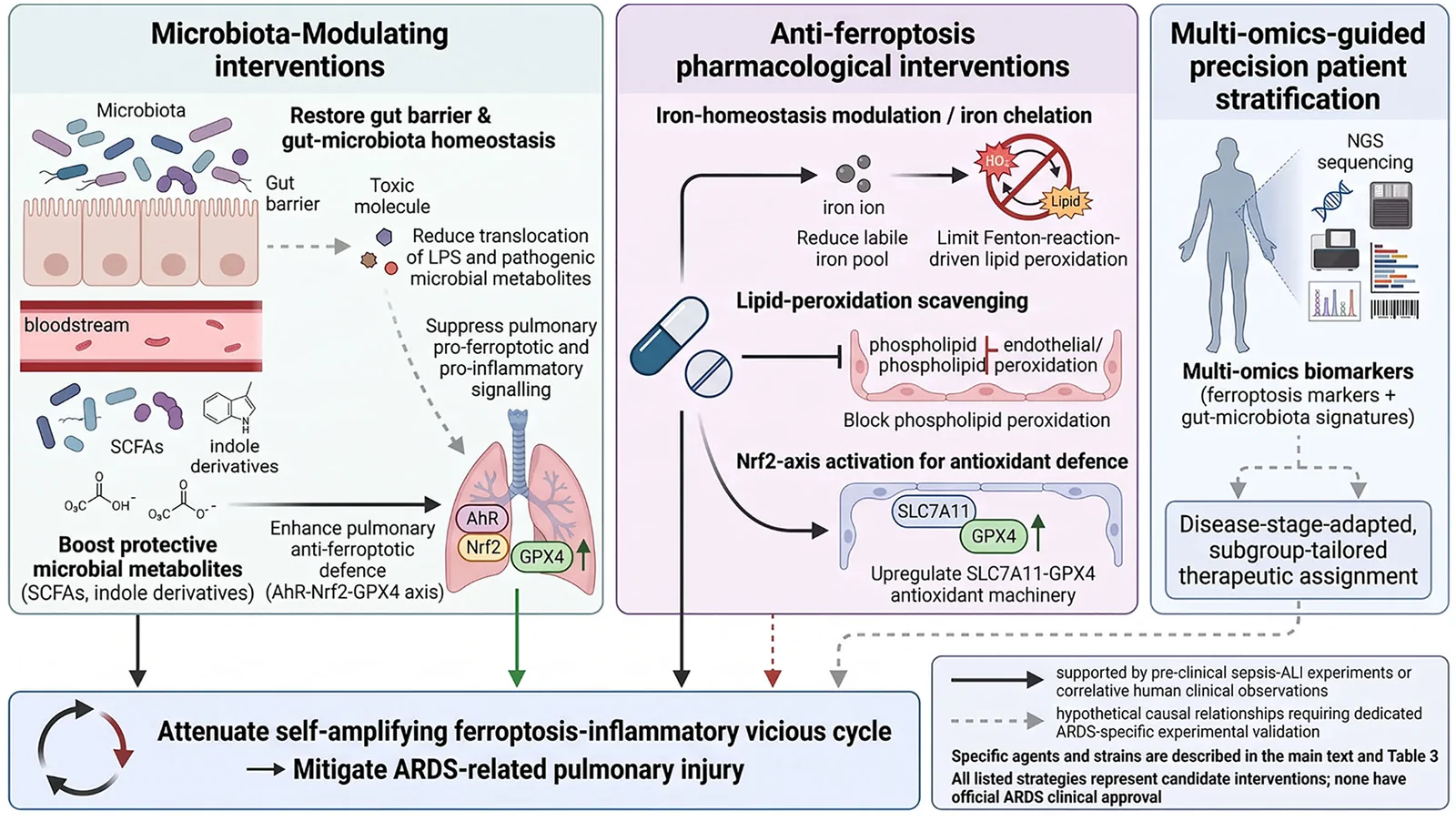
Summary of candidate therapeutic strategies targeting the gut-microbiota-ferroptosis-immune loop in ARDS. Solid black lines: interventions/effects supported by pre-clinical sepsis-ALI experiments or correlative human clinical observations. Dashed grey lines: hypothetical causal relationships requiring dedicated ARDS-specific experimental validation. This schematic displays only high-level therapeutic strategy categories. Details of small-molecule agents, microbial strains and references are provided in the main text and **Table 3** to reduce visual complexity. All strategies represent candidate interventions and lack official clinical approval for ARDS.

Literature search strategy: We searched PubMed, Web of Science and Embase for articles published up to submission date (October 2026). Landmark foundational publications are retained irrespective of publication year for conceptual background. Search terms included “ARDS”, “acute lung injury”, “ferroptosis”, “gut microbiota”, “gut-lung axis”, “microbial metabolites”, “bile acids”, “short-chain fatty acids”, “indole derivatives”, “macrophage polarization”, “neutrophil extracellular traps”. Primary mechanistic original articles, clinical cohort studies and relevant reviews were prioritized. Evidence was categorized as direct human ARDS evidence, ARDS/ALI animal evidence, sepsis-related pulmonary-model evidence, or non-pulmonary extrapolation. Non-English publications were excluded. This is a narrative review without formal PRISMA compliance.

## 2 Ferroptosis as an immunometabolic driver in ARDS

This chapter delves into intrinsic, lung-local pathological consequences driven by ferroptosis in ARDS. It focuses on pulmonary pathological outcomes triggered by ferroptosis: ferroptosis reshapes macrophage metabolism and polarization, which in turn fuels self-amplifying alveolar inflammatory damage. In this context, ferroptosis emerges as a key damage initiator that dynamically modulates the functional landscape of lung innate and adaptive immune populations—particularly macrophages, neutrophils, and T lymphocytes—and concurrently sustains a self-reinforcing interplay between ferroptotic events and inflammatory cascades.

Ferroptosis has emerged as a pivotal form of iron-dependent regulated cell death that drives acute lung damage in ARDS. It is distinguished by unrestrained iron overload, excessive lipid peroxidation, and collapse of the antioxidant system governed by glutathione peroxidase 4 (GPX4), ultimately leading to catastrophic membrane injury in alveolar epithelial and endothelial cells (32–35). In ARDS, these features directly impair immune cell function and fuel a self-sustaining inflammatory cascade.

### 2.1 Molecular mechanisms of ferroptosis

Abnormal iron metabolism serves as a primary trigger of ferroptosis in ARDS. NCOA4-mediated ferritinophagy promotes labile iron release and lipid peroxidation in lung tissues. Hepcidin/HAMP, in contrast, may modulate ferritin-dependent iron sequestration and has been reported to alleviate LPS-induced ARDS in preclinical models, although its role in human ARDS remains context-dependent (33, 34, 36). Hepcidin is an iron-homeostasis-regulating peptide; it modulates ferritin-dependent iron sequestration but is not an upstream inducer of NCOA4-mediated ferritinophagy. The resulting iron overload fuels Fenton reactions, generating massive reactive oxygen species (ROS) that initiate lipid peroxidation and cytotoxicity. This iron-dependent oxidative damage directly contributes to ferroptotic death in pulmonary epithelial and endothelial cells during sepsis-associated ARDS (37, 38).

Dysregulated lipid metabolism and antioxidant failure further amplify ferroptosis. Activation of lipoxygenase (LOX) and acyl-CoA synthetase long-chain family member 4 (ACSL4) accelerates oxidation of polyunsaturated fatty acids (PUFAs), producing toxic lipid peroxides such as malondialdehyde (MDA) and 4-hydroxynonenal (4-HNE) (32–34). Meanwhile, suppression of the Nrf2/GPX4 antioxidant axis compromises cellular peroxide-clearing activity, exacerbating oxidative injury. Nrf2 mitigates ferroptosis by upregulating downstream targets including HO-1 and SLC7A11, thereby protecting against alveolar damage in ARDS (39–41).

Ferroptosis propagates lung injury by fueling a self-sustaining inflammatory cascade. Damage-associated molecular patterns (DAMPs) released by ferroptotic cells activate macrophages and neutrophils, triggering secretion of interleukin-6 (IL-6), tumor necrosis factor-α (TNF-α), and other pro-inflammatory cytokines (42–45). This “ferroptosis–inflammation cycle” disrupts the alveolar-capillary barrier, aggravates pulmonary edema, and exacerbates ARDS progression. Moreover, systemic cytokine storms elicited by sepsis or viral infection may further amplify ferroptosis-related NF-κB/MAPK signaling, forming a vicious pathological loop (46).

Ferroptosis also crosstalks with other cell death modalities in ARDS. Ferroptosis and pyroptosis mutually reinforce each other via shared ROS signaling; lipid peroxides activate the NOD-, LRR- and pyrin domain-containing protein 3 (NLRP3) inflammasome to promote IL-1β maturation and inflammatory amplification (47). Crosstalk with necroptosis may also contribute to alveolar epithelial cell death, although the interaction between the RIPK1/RIPK3 pathway and ferroptosis remains to be fully clarified (47, 48).

### 2.2 Macrophage polarization and ferroptosis

In preclinical ARDS models, macrophage polarization, metabolic reprogramming, and ferroptosis form a tightly interconnected and synergistic network driven by iron dyshomeostasis, oxidative stress, and altered immunometabolic signaling (49, 50). Iron overload activates the nuclear factor-κB (NF-κB)/hypoxia-inducible factor 1-alpha (HIF-1α) axis and may contribute to pro-inflammatory macrophage-state polarization while suppressing repair-associated phenotypes, thereby may amplify inflammatory injury; these observations are largely derived from non-pulmonary models and await dedicated validation in ARDS alveolar macrophages (51, 52). Concurrently, nuclear receptor coactivator 4 (NCOA4)-mediated ferritinophagy releases labile free iron, which aggravates lipid peroxidation and initiates the ferroptotic cascade (53–55). This process is accompanied by DAMPs such as HMGB1 and altered hepcidin-related iron handling, which may contribute to a positive ferroptosis-inflammation feedback loop; direct ARDS-specific evidence is limited (14, 56).

Notably, macrophage functional phenotype is profoundly coupled with cellular metabolic fate and ferroptosis susceptibility. Human ARDS alveolar macrophages exhibit heterogeneous activation states that cannot be fully captured by the simplified M1-M2 binary classification framework; such binary nomenclature originates largely from *in-vitro* and pre-clinical animal models. Observations of state-dependent ferroptosis susceptibility are largely inferred from non-pulmonary pre-clinical models and remain to be directly validated in human ARDS samples. In pre-clinical model systems, pro-inflammatory macrophage states remodel the local metabolic microenvironment through glycolysis and massive reactive oxygen species (ROS) burst, further amplifying lipid peroxidation and ferroptotic cell death (49, 51). In contrast, repair-associated macrophage states activate antioxidant defense pathways—including the Nrf2/HO-1 axis—through oxidative phosphorylation (OXPHOS) and fatty acid oxidation, thereby conferring relative resistance to ferroptosis (57). Under specific iron-overload or p62/SQSTM1-dependent conditions, even repair-associated macrophage populations may become more vulnerable to ferroptosis, as demonstrated in non-pulmonary models (58). Such state-dependent differences in ferroptosis susceptibility can produce a skewed pro-inflammatory milieu that amplifies cytokine release (e.g., IL-6, TNF-α) and further sensitizes neighboring epithelial and endothelial cells to ferroptosis, thereby accelerating alveolar-capillary damage in ARDS (2, 51, 59).

From a mechanistic standpoint, interrupting this ferroptosis-polarization feedback loop and shifting macrophage states away from pro-inflammatory programs represents a promising conceptual therapeutic direction. Key regulatory nodes within this circuit include the Nrf2-ferroptosis axis (60), PI3K/AKT (61), TNF-related signalling (62), and MAPK1/ROS pathways (63). Detailed discussion of pharmacological candidates targeting these nodes is deferred to Chapter 5.

### 2.3 Neutrophil extracellular traps and ferroptosis

In animal studies of sepsis and ARDS, neutrophil extracellular trap (NET) formation requires iron and ROS. Iron overload enhances mitochondrial oxidative stress and chromatin decondensation, thereby promoting NET release (64, 65). During sepsis, platelet-derived iron triggers ferroptosis-dependent NETosis, further aggravating endothelial ferroptosis and organ damage (64, 66). NETs induce ferroptosis in vascular cells by depleting mitochondrial glutathione (mitoGSH), while DNase I-mediated NET inhibition alleviates cytotoxicity (65–67). Histones and myeloperoxidase (MPO) released by NETs can also directly activate ferroptotic signaling in adjacent cells (65, 66). Consequently, NET-related ferroptosis may contribute to disruption of the alveolar-capillary barrier, promoting edema and inflammatory cell extravasation, which may perpetuate the ferroptosis-inflammation cycle in ARDS.

NETs form a complex regulatory network with macrophage polarization and ferroptosis. HMGB1 released by NETs activates TLR4 signaling in macrophages, driving pro-inflammatory macrophage states and suppressing repair-associated macrophage functions, while inducing macrophage ferroptosis via iron and ROS to impair barrier integrity (68, 69). In pathological settings, NETs promote inflammation and tissue damage by weakening macrophage phagocytosis and inducing ferroptosis; DNase I-mediated NET degradation restores immune balance (70–72). Thus, targeting NET formation (e.g., with DNase I or iron chelators) simultaneously curbs macrophage ferroptosis and favours a shift away from excessive pro-inflammatory macrophage activity, offering a dual immunomodulatory strategy for ARDS.

Imbalanced macrophage polarization and iron-dependent NET formation drive acute inflammatory lung injury. Hepatic IRI studies reveal that NETs trigger macrophage ferroptosis (73, 74), but this paradigm requires verification in ARDS alveolar immune cells. These observations are largely derived from non-ARDS or non-pulmonary experimental systems and serve only as hypothesis-generating evidence, rather than established ARDS pathogenic mechanisms. (Non-pulmonary extrapolation). Further targeted *in vivo* and *in vitro* ARDS studies are needed to confirm whether blocking NET-induced macrophage ferroptosis can mitigate pulmonary inflammatory injury and protect alveolar structure (75, 76).

### 2.4 T-cell ferroptosis and adaptive immunity

Ferroptosis is well-established as a driver of alveolar epithelial and endothelial injury in ARDS (77). These observations regarding T-cell-related ferroptosis are largely derived from non-ARDS or non-pulmonary experimental systems and serve only as hypothesis-generating evidence, rather than established ARDS pathogenic mechanisms. Most mechanistic data on T-cell ferroptosis derive from non-pulmonary disease models (e.g., cancer, autoimmune disease) (Non-pulmonary extrapolation).

ARDS-Specific Ferroptosis Evidence.

Multiple ARDS-focused studies confirm ferroptosis mediates acute lung damage. SERPINE1 rises in ARDS patients and LPS-challenged alveolar cells, exacerbating ferroptosis through the SERPINE1-NAD/Sirt3 cascade; inhibiting this pathway restores SLC7A11/GPX4 and alleviates lung injury (2). Sepsis-related ARDS also features disordered iron metabolism and lactate-mediated endothelial ferroptosis. Dipyridamole exerts anti-ferroptotic protective effects in preclinical ARDS models and preliminary clinical cohorts via multiple molecular mechanisms, which are detailed in Section 5.1 (12). Additional evidence including circulating miR-744 and α7nAchR-targeted electroacupuncture further validates ferroptosis as a therapeutic target in lung injury (42, 78).

### 2.5 Balancing inflammation and antioxidant defenses

The progression of ferroptosis-associated lung injury is governed by the dynamic equilibrium between pro-inflammatory TLR4/NLRP3 cascades and the cytoprotective Nrf2 antioxidant axis, which dictates the severity of the self-amplifying ferroptosis-inflammation vicious cycle disrupting pulmonary immune homeostasis (79, 80). Sustained GPX4 activity is the core checkpoint to interrupt oxidative and inflammatory positive feedback loops. Relevant regulatory evidence documented in non-pulmonary experimental systems still lacks verification in ARDS-specific preclinical and clinical investigations (81–86). In brief, three major regulatory axes shape intrinsic pulmonary ferroptosis susceptibility: the anti-ferroptotic AhR/Nrf2 axis, pro-ferroptotic TLR4/NF-κB axis, and bile-acid-responsive FXR/TGR5 balancing hub. This chapter focuses on intrinsic pulmonary cell-level pathological machinery, without discussing gut-derived metabolite inputs. This section provides only high-level conceptual labels and omits full intracellular-cascade descriptions. Pulmonary phenotypic outcomes triggered by gut-derived microbial metabolites are elaborated in Chapter 4.

The core mechanisms of ferroptosis and their interaction with immune cells in ARDS are summarized schematically in **Figure 2**.

## 3 Gut microbiota–gut–lung axis: bidirectional immune regulation and evidence gaps in ARDS

Having established in Chapter 2 that ferroptosis-driven immune dysregulation exacerbates lung injury in ARDS, we now examine the putative pulmonary-to-gut limb of the bidirectional gut–lung axis. Within our hypothetical temporal model, intestinal dysbiosis is considered a putative downstream pathological consequence that may be triggered by ARDS-associated pulmonary insult. Definitive causal evidence for this pulmonary-driving effect remains lacking. This chapter summarizes correlative clinical and pre-clinical observations, which provide indirect background for metabolite-driven pulmonary modulation elaborated in Chapter 4. We specifically address: (i) proposed mechanisms by which pulmonary injury may disturb intestinal homeostasis; (ii) ARDS-associated gut dysbiosis and its ICU-related confounders; (iii) gut-to-lung immune regulation; and (iv) how the full feed-forward loop can be experimentally tested.

### 3.1 Proposed lung-to-gut mechanisms and evidence gaps

#### 3.1.1 ICU-related clinical confounders for interpreting gut dysbiosis in ARDS

Notably, multiple ICU-relevant clinical confounders can independently reshape gut microbiota profiles in ARDS patients and complicate causal inference for the pulmonary-to-gut cascade. These confounders include broad-spectrum antibiotics (87), sedative agents (88), gastric acid-suppressing medications (89), modes of enteral nutrition (90), vasopressor therapy, and systemic stress induced by mechanical ventilation (8). Such interventions frequently alter gut community composition independent of pulmonary-derived inflammatory signals.

It should be emphasized that the Phase 1a→1b→2 cascade represents an etiology-dependent working hypothesis rather than a universal pathogenic sequence for all ARDS subtypes (see **Box 1** for full definition of Phase 1a-1b-2 working model). In indirect/sepsis-associated ARDS, intestinal barrier dysfunction and gut dysbiosis can occur prior to or concurrently with pulmonary injury and are not necessarily secondary consequences of lung damage. Several plausible mechanistic pathways have been postulated to explain how pulmonary injury might secondarily disturb intestinal homeostasis. These include systemic overflow of pro-inflammatory cytokines (e.g., TNF-α, IL-6, IL-1β), splanchnic hypoperfusion and intestinal ischemia-reperfusion injury, as well as excessive sympathetic activation that may disrupt epithelial tight-junction integrity (occludin, claudin-1, ZO-1). Hypoxia secondary to respiratory failure can also impair intestinal epithelial repair capacity and increase epithelial permeability. In addition, mechanical ventilation, vasopressor therapy and enteral feeding intolerance may indirectly compromise intestinal perfusion and barrier function (1, 11).

Nevertheless, the majority of these mechanistic observations are extrapolated from general sepsis, hemorrhage and ischemia-reperfusion animal models. Dedicated ARDS-specific *in vivo* evidence showing that isolated pulmonary injury sequentially disrupts gut-barrier function remains sparse. Most clinical studies report cross-sectional associations between ARDS severity and gut microbial alterations, without establishing temporal precedence or directionality. Therefore, the pulmonary-to-gut limb should be regarded as a mechanistically plausible but unproven component of the proposed framework (1, 3).

It should be noted that changes in gut-microbiota profiles observed in ARDS patients may plausibly result from multiple ICU-related interventions independent of pulmonary-origin injury. Observed gut dysbiosis in ARDS cohorts cannot be solely attributed to pulmonary injury-related mechanisms (1).

### 3.2 ARDS-associated gut dysbiosis: associations and confounders

ARDS patients exhibit marked alterations in gut-microbiota composition, which are shaped jointly by pulmonary inflammation and well-recognized ICU-related confounding exposures (see Section 3.1.1). Clinical studies and animal experiments consistently reveal that ARDS patients exhibit elevated abundance of Gram-negative bacteria (e.g., Enterobacteriaceae) and reduced beneficial microbes (e.g., Lactobacillus, Bifidobacterium), closely associated with impaired intestinal mucosal barrier function (29, 91, 92). In sepsis-associated ARDS, gut microbiota can even translocate to the lungs, as evidenced by increased intestinal flora proportions in bronchoalveolar lavage fluid (93). Moreover, ARDS disturbs the profiles of gut microbial metabolites — such as decreased short-chain fatty acids (SCFAs) — via oxidative stress and inflammatory cytokine release, further exacerbating intestinal microecological disorders (29). The characteristic changes in gut microbial composition in ARDS and their functional impacts are summarized in **Table 1**.

**Table 1:** Gut microbiota changes in ARDS and functional impacts.

Metabolic taxa change	Direction	Functional impact/Mechanism	Proposed contribution/observed association with ARDS progression
Lactobacillus, Faecalibacterium (Firmicutes)	Decreased	Reduced production of SCFAs and other protective metabolites; impaired intestinal barrier integrity	May exacerbate systemic inflammation and pulmonary injury
Bifidobacterium (Actinobacteria)	Decreased	Reduced production of SCFAs and other protective metabolites; impaired intestinal barrier integrity	May exacerbate systemic inflammation and pulmonary injury
Enterobacteriaceae, Clostridium, Klebsiella	Increased	Enhanced LPS release; activation of TLR4/NF-κB signaling; promotion of inflammatory cytokine production	May aggravate alveolar damage and ferroptosis
Gut microbial diversity	Decreased	Loss of metabolic and immune homeostasis; reduced resistance to pathogens	May promote dysbiosis-driven lung injury
Intestinal barrier function	Impaired	Increased gut permeability; translocation of bacteria and endotoxins to the circulation and lungs	May fuel systemic inflammation and ARDS severity

Data summarized and compiled from references (6, 7, 29, 91–93, 96, 99, 100). Bifidobacterium belongs to phylum Actinobacteria and is listed separately from the Firmicutes taxa.

### 3.3 Immune regulation by gut microbiota: gut-to-lung evidence

Conversely, gut microbiota may modulate ARDS progression through two major pathways. First, immune regulation: microbial dysbiosis has been associated with aberrant Toll-like receptor signaling, T helper 17 (Th17)/Treg imbalance and aggravated pulmonary inflammation (29, 94). Antibiotic-mediated depletion of gut microbiota may worsen lung injury by attenuating these immunoregulatory effects in preclinical models (95). Second, protective effects mediated by microbial metabolites: SCFAs (e.g., butyrate) produced by microbial fermentation may alleviate oxidative stress and inhibit the NF-κB pathway. Additionally, compounds such as resveratrol have been reported to attenuate ARDS by enriching beneficial bacteria including Lactobacillus reuteri (94, 96).

The gut–lung axis operates through immune-mediated and metabolite-mediated routes in a bidirectional way. On one hand, pulmonary inflammatory disturbance releases proinflammatory cytokines (e.g., IL-6, TNF-α) that may disrupt intestinal immune homeostasis. On the other hand, gut microbiota feedback-modulates pulmonary inflammation by regulating systemic immune responses such as macrophage polarization (97–99).

### 3.4 How to test the feed-forward loop: an open research question

Most available pre-clinical studies administer microbiota-related interventions at the time of lung-injury induction; such designs test preventive or simultaneous protective effects rather than answering whether correcting gut disturbance can ameliorate already-established pulmonary injury after ARDS onset. Rigorous validation of the full Phase 1a–1b–2 feed-forward cascade requires temporally separated experimental approaches. In pre-clinical animal models: pulmonary injury should be fully established first, followed by delayed manipulation of gut barrier or gut microbiota (e.g., delayed FMT, metabolite supplementation, gut-barrier protective agents). Serial sampling should cover gut-barrier biomarkers (IFABP, tight-junction proteins), gut microbial profiles, circulating metabolites, and sequential pulmonary ferroptosis-associated readouts (12). For human ARDS research: longitudinal multi-omics sampling is required, with statistical adjustment for ICU confounders(detailed in Section 3.1.1) including antibiotic exposure, acid-suppressive medication, sedation and enteral feeding (1). Mediation-analysis approaches may help disentangle whether gut-microbial signatures act as true mediators of pulmonary deterioration. Ex vivo gut–lung microphysiological chip systems can further assist mechanistic deconvolution. Collectively, the Phase 1a–1b–2 cascade (see **Box 1** for full definition of Phase 1a-1b-2 working model) should be treated as a testable working hypothesis instead of an established pathogenic sequence (1).

## 4 Gut-derived microbial metabolites and their potential modulation of pulmonary ferroptosis in ARDS

### 4.1 Gut microbial phyla and their ferroptosis-modulating metabolites

ARDS-associated gut dysbiosis is characterized by reproducible shifts at the phylum level, and each major phylum is associated with a distinct metabolite profile that can either restrain or promote pulmonary ferroptosis. This phylum-level overview provides a high-level conceptual framework; ARDS-relevant cellular phenotypes and experimental evidence are elaborated in Sections 4.2-4.5 below.

#### 4.1.1 Firmicutes and associated metabolites: predominantly protective in current preclinical models

These phylum-level shifts represent coarse ecological correlates; functional outcomes are highly species-, strain-, substrate- and host-context-dependent. Key Firmicutes genera: Faecalibacterium, Clostridium, Lactobacillus. Bifidobacterium (phylum Actinobacteria) is discussed separately because it also produces SCFAs and supports intestinal barrier integrity. Primary metabolites: Short-chain fatty acids (SCFAs like butyrate, propionate, and acetate) and tryptophan-derived indoles. Main signaling axis: AhR/Nrf2-SLC7A11-GPX4 (Key background regulatory principles of these signalling axes are summarized in Section 2.5; detailed metabolite-triggered pulmonary phenotypic outputs are elaborated below).

The commensal Faecalibacterium prausnitzii, as a primary butyrate producer, is markedly depleted in ARDS patients and animal models, a reduction that correlates with exacerbated alveolar ferroptosis and sustained inflammatory injury (29). Butyrate and other SCFAs derived from Firmicutes-mediated fermentation confer pulmonary anti-ferroptotic and tissue-protective phenotypes that attenuate acute lung injury (ALI) (26, 100). Tryptophan-metabolizing Clostridium species convert intestinal tryptophan into indole derivatives (e.g., indole-3-propionic acid and indole-3-aldehyde), which translocate to the lung and restrain pro-ferroptotic macrophage-driven inflammatory profiles and cellular ferroptosis vulnerability (27, 29, 101). Lactobacillus and Bifidobacterium exert dual protective functions by reinforcing the intestinal barrier to block systemic lipopolysaccharide (LPS) translocation and by secreting SCFAs that reduce iron uptake in pulmonary immune cells (30, 102). Clinical and preclinical ARDS phenotype: A marked reduction in Firmicutes abundance correlates with impaired endogenous antioxidant signaling and aggravated alveolar ferroptosis in ARDS pre-clinical models and clinical cohorts, although causal inference remains difficult, consistent with the microbial alterations listed in **Table 1** (2, 29).

#### 4.1.2 Bacteroidetes: a phylum with biphasic regulatory roles in ferroptosis

##### 4.1.2.1 Representative genus: bacteroides

Main signaling axes: AhR/Nrf2 (anti-ferroptotic) and FXR/TGR5 (pro-ferroptotic) (Key background regulatory principles of these signalling axes are summarized in Section 2.5; detailed metabolite-triggered pulmonary phenotypic outputs are elaborated below).

This phylum produces two distinct classes of metabolites that exert contrasting effects on ferroptotic pathways. On one hand, tryptophan-derived indoles act as anti-ferroptotic agents. Commensal strains such as Bacteroides uniformis sustain intestinal tryptophan catabolism, yielding indole derivatives that shield macrophages from ferroptotic death (29, 101). On the other hand, conversion of primary bile acids leads to accumulation of conjugated primary bile acids such as glycochenodeoxycholic acid (GCDCA), together with altered pools of secondary bile acids such as DCA and LCA that have been proposed to amplify iron uptake and lipid peroxidation within lung macrophages in non-pulmonary models (36, 103). The overabundance of pathogenic Bacteroides vulgatus is linked to elevated bile salt hydrolase activity, accelerating the biotransformation of primary bile acids into toxic secondary forms. For instance, circulating GCDCA has been hypothesized to exacerbate pulmonary ferroptotic injury.

Fundamentally, Bacteroidetes does not exert a monolithic protective or detrimental effect. The ultimate impact on ferroptosis hinges on the equilibrium between tryptophan degradation and bile acid modification, a dynamic that likely accounts for the contradictory outcomes observed in prior *in vivo* experiments (27, 29, 36, 101, 103).

#### 4.1.3 Proteobacteria and LPS: potential pro-ferroptotic signals in ARDS

Representative family: Enterobacteriaceae; representative genera: Klebsiella, Escherichia. Core microbial structural product/endotoxin: Lipopolysaccharide (LPS), an outer-membrane endotoxin. Targeted signaling axis: TLR4/NF-κB. (Key background regulatory principles of these signalling axes are summarized in Section 2.5; detailed pulmonary phenotypic outputs triggered by gut-derived microbial metabolites and structural products are elaborated below).

Compromise of the intestinal barrier may permit the unchecked proliferation of Proteobacteria and the massive release of LPS, which translocates systemically via the gut-lung axis (26, 29, 101, 104). Systemically circulating LPS may trigger pro-inflammatory and pro-ferroptotic cellular phenotypes in the ARDS lung, favouring glycolytic M1 macrophage polarization, culminating in a massive reactive oxygen species (ROS) burst and subsequent ferroptosis in both alveolar and immune cells (26, 29, 101).

Clinical relevance in ARDS: Expansion of Proteobacteria has been associated with severe ARDS in several cohorts, but whether it is a causal driver or a severity-associated marker remains unclear. Its abundance positively correlates with elevated malondialdehyde (MDA) levels in bronchoalveolar lavage fluid (BALF), indicating intensified lipid peroxidation and ferroptotic damage in the lungs (2, 29, 35, 104).

#### 4.1.4 Phylum-based regulatory network of ferroptosis

The Firmicutes/Bacteroidetes ratio is a crude ecological correlate in critically-ill cohorts, but it lacks sufficient robustness to carry mechanistic weight for explaining ferroptosis susceptibility in ARDS:

Physiological steady state: High Firmicutes abundance + moderate Bacteroides levels → sufficient SCFA and indole production, balanced bile acid metabolism → intact GPX4 antioxidant capacity, low pulmonary ferroptosis risk.

ARDS dysbiotic state: Firmicutes depletion + Proteobacteria overgrowth + disrupted bile acid metabolism in Bacteroides → impaired AhR/Nrf2 activity, hyperactive TLR4/FXR pro-ferroptotic cascades → sustained alveolar ferroptosis and self-amplifying inflammatory loops.

All microbiota-metabolite-ferroptosis signaling chains elaborated in this section fully correspond to the four classes of gut-derived metabolites summarized in **Table 2**, consolidating scattered mechanistic evidence into an integrative overview.

**Table 2:** Gut microbial metabolites and structural products and their proposed/observed modulation of pulmonary ferroptosis in ARDS.

Metabolite class/structural product	Gut microbial source	Core regulatory principles (summarized in Section 2.5)	Proposed/observed pulmonary phenotype	Evidence level	References
Short-chain fatty acids (butyrate, propionate, acetate)	Firmicutes (Faecalibacterium, Clostridium, Lactobacillus)	Anti-ferroptotic; AhR/Nrf2-dependent signalling	May raise the ferroptosis threshold in alveolar macrophages and type-II alveolar epithelial cells, elevate GSH, attenuate lipid peroxidation, and reduce acute lung injury severity	Sepsis-ALI pre-clinical; partial ARDS clinical correlation	(4, 29, 99–101)
Indole derivatives (indole-3-propionic acid, indole-3-aldehyde, indole-3-acetic acid)	Bacteroidetes and tryptophan-metabolizing Clostridium	Anti-ferroptotic; AhR/Nrf2-dependent signalling	May favour anti-inflammatory M2 macrophage polarization, lower IL-1β output, reduce pulmonary lipid peroxidation, improve gas exchange, and stabilize the intestinal barrier to limit LPS translocation	Sepsis-ALI pre-clinical; partial ARDS clinical correlation	(29, 99, 101, 103, 107)
Lipopolysaccharide (LPS)	Proteobacteria (Klebsiella, Escherichia)	Pro-ferroptotic; TLR4/NF-κB-dependent signalling	May drive glycolytic pro-inflammatory M1 macrophage polarization, massive ROS burst, ferroptotic death of alveolar and immune cells, NETosis and endothelial ferroptosis with vascular leakage	Sepsis-ALI pre-clinical	(38, 43, 99, 102, 104)
Conjugated primary bile acids (GCDCA)	Bacteroides (e.g., B. vulgatus)	Pro-ferroptotic; bile-acid–FXR/TGR5-responsive signalling	May increase iron uptake and lipid peroxidation in lung macrophages, shift cells toward pro-ferroptotic states	Non-pulmonary extrapolation; Hypothetical	(106, 109, 114, 116, 117, 125)
Secondary bile acids (DCA, LCA)	Bacteroides (e.g., B. vulgatus); bile-salt-hydrolase-active taxa	Pro-ferroptotic; bile-acid–FXR/TGR5-responsive signalling	May increase iron uptake and lipid peroxidation in lung macrophages; loss of protective UDCA may further aggravate this pathological shift	Non-pulmonary extrapolation; Hypothetical	(106, 109, 114, 116, 117, 125)

Key background regulatory principles of these signalling axes are summarized in Section 2.5; this table focuses exclusively on proposed/observed pulmonary phenotypes. Signalling-cascade mechanistic details (including ACSL4, TFR1, SLC7A11 and GPX4 regulatory events) are not expanded within this table.

Evidence-level classification: Human ARDS data, human ARDS-specific clinical or ex-vivo studies; ARDS/ALI pre-clinical, ARDS/ALI experimental animals; Sepsis-ALI pre-clinical, sepsis-associated acute lung-injury animal models; Non-pulmonary extrapolation, observations obtained from non-respiratory disease models; Hypothetical, mechanistic inference without direct experimental validation. Combined entries formerly using “/” are separated by semicolons to list each evidence category independently.

Note: Most metabolite-driven pulmonary phenotypic effects described throughout Sections 4.2-4.5 are derived from pre-clinical or non-pulmonary models; dedicated ARDS-specific *in-vivo* human validation remains largely absent.

### 4.2 Protective gut microbial metabolites: SCFAs and indole derivatives acting on the AhR/Nrf2 signaling node

Building on the phylum-level overview in Section 4.1, we next discuss potential ARDS-relevant pulmonary phenotypes inferred from indirect pre-clinical evidence for these metabolites (105, 106). Here we focus specifically on how gut-derived protective metabolites—SCFAs from Firmicutes and indole derivatives from Bacteroidetes—engage this pre-existing pulmonary signaling node to restrain ferroptosis in ARDS.

#### 4.2.1 SCFAs: pulmonary protective phenotypes triggered by Firmicutes-derived short-chain fatty acids

Butyrate, propionate, and acetate reach the lung via the systemic circulation after intestinal absorption. Following exposure to these Firmicutes-derived metabolites, alveolar macrophages and type-II pulmonary epithelial cells exhibit a raised cellular ferroptosis threshold, accompanied by attenuated lipid peroxidation within ARDS-affected pulmonary tissue. Propionate- and acetate-triggered immune-cell responses further limit pro-ferroptotic cellular phenotypes. Collectively, SCFA exposure may confer anti-ferroptotic, tissue-protective outcomes in the ARDS lung.

#### 4.2.2 Indole derivatives: anti-ferroptotic outcomes of tryptophan-derived microbial metabolites

Indole-3-propionic acid (IPA), indole-3-aldehyde (IAld), and indole-3-acetic acid (IAA) may engage pulmonary AhR/Nrf2 axis to exert putative anti-ferroptosis effects in ARDS. Direct experimental evidence from ARDS-specific *in-vivo* studies is still limited.These microbial metabolites confer anti-inflammatory phenotypes in alveolar macrophages, favouring repair-associated macrophage states and lowered IL-1β output, while stabilizing intestinal tight junctions to limit endotoxin translocation. Indole metabolites secreted by Bacteroides further sustain cellular redox balance and restrain pro-inflammatory cellular states (107).

IAld, IAA, and IPA shape the responses of pulmonary macrophages and endothelial cells. These indole-derived metabolites reduce pulmonary ferroptotic injury in ARDS-relevant cellular contexts. Indole derivatives also reduce intestinal permeability by upregulating tight-junction proteins (occludin, claudin-1) in the gut epithelium, which secondarily limits the entry of pro-ferroptotic LPS into the circulation. In pre-clinical studies, supplementation with indole-3-carbinol or IPA has been proposed to reduce pulmonary lipid peroxidation and improve gas-exchange outcomes, yet robust ARDS-specific *in-vivo* validation remains lacking (sepsis-ALI pre-clinical/non-pulmonary extrapolation).

#### 4.2.3 Synergy between SCFAs and indole metabolites

SCFAs and indole metabolites exert complementary protective effects upon the same anti-ferroptotic signaling node. The simultaneous decline of both metabolite classes in ARDS-associated dysbiosis therefore produces a compounded loss of anti-ferroptotic defense, rendering pulmonary cells more susceptible to iron-dependent cell death.

We hypothesize that this metabolite-driven protective state can suppress NET formation and break the ferroptosis-inflammatory vicious cycle in ARDS; dedicated sepsis-ALI animal and primary alveolar cell experiments are required to verify this conjecture. Related protective effects observed in intestinal ischemia-reperfusion models only serve as secondary minor references and will not be elaborated herein.

### 4.3 Pathogenic gut-derived microbial metabolites and structural products: LPS and bile acids acting on the TLR4/NF-κB signaling node

Building on the phylum-level overview in Section 4.1, we next discuss potential ARDS-relevant pulmonary phenotypes inferred from indirect pre-clinical evidence for these metabolites (104, 108–110). Here we focus specifically on how gut-derived pathogenic metabolites—LPS from expanded Proteobacteria and dysregulated bile acids such as GCDCA—engage this pre-existing pulmonary signaling node to promote ferroptosis in ARDS.

#### 4.3.1 LPS: pro-ferroptotic pulmonary phenotypes induced by Proteobacteria-derived endotoxin

As a gut-derived pro-ferroptotic mediator in ARDS, circulating LPS engages pulmonary TLR4/NF-κB signalling and may drive robust inflammatory-oxidative phenotypes in pulmonary macrophages (104, 108), triggering high-level pro-inflammatory cytokine release and mitochondrial dysfunction that accelerates ferroptosis (2, 111).

LPS translocates across the compromised intestinal barrier in ARDS and reaches the lung via the portal and systemic circulation (104). Upon exposure to this gut-derived endotoxin, ARDS pulmonary macrophages exhibit prominent pro-ferroptotic and pro-inflammatory cellular phenotypes (78). This exposure may establish an autocrine-paracrine feedforward inflammatory-ferroptotic loop within the lung (2). In pulmonary endothelial cells, gut-derived LPS challenge additionally promotes NETosis and endothelial ferroptosis, exacerbating vascular leakage (see Section 2.3 for NET–ferroptosis crosstalk) (65, 66).

#### 4.3.2 GCDCA and dysregulated bile-acid pools: pathological cellular outcomes in ARDS lungs

Gut dysbiosis in ARDS alters the microbial biotransformation of primary bile acids, leading to accumulation of conjugated primary bile acids such as glycochenodeoxycholic acid (GCDCA) and a reduction in beneficial secondary bile acids such as ursodeoxycholic acid (UDCA). After pulmonary macrophage exposure to GCDCA, ARDS-relevant inflammatory microenvironments may shift toward pro-ferroptotic cellular characteristics. It should be noted that bile-acid species exert context-dependent effects on ferroptosis; FXR activation has been reported to suppress lipid peroxidation, whereas other bile-acid pools may promote iron-dependent injury in specific non-pulmonary models (109, 110). Direct ARDS lung-specific experimental evidence remains lacking. The concurrent loss of protective UDCA may further worsen this pathological shift.

#### 4.3.3 Convergence of LPS and bile acid signaling on pulmonary ferroptosis

LPS and GCDCA converge on the same downstream pro-ferroptotic node. Their simultaneous elevation in ARDS-associated dysbiosis therefore may produce a synergistic pro-ferroptotic effect that exceeds the impact of either metabolite alone (109, 110). This LPS-GCDCA synergistic pathogenic loop in ARDS requires dedicated lung-specific animal and cellular validation. Related observations in extra-pulmonary tumors and metabolic disorders are not elaborated here.

### 4.4 Bile acid–FXR/TGR5 signaling as a modulator of ferroptosis susceptibility in ARDS

Building on the phylum-level overview in Section 4.1, we next discuss potential ARDS-relevant pulmonary phenotypes inferred from indirect pre-clinical evidence for these metabolites (105). Specifically, we focus on how specific gut microbial metabolites—particularly bile acids derived from Bacteroides species—shape pulmonary cell-type-specific ferroptotic susceptibility within the ARDS lung.

#### 4.4.1 Bacteroides-derived secondary bile acids as key modulators of FXR/TGR5

Observations from non-pulmonary pharmacological and genetic-manipulation studies provide indirect context for these phenotypic shifts (110, 112). These observations are largely derived from non-ARDS or non-pulmonary experimental systems and serve only as hypothesis-generating evidence, rather than established ARDS pathogenic mechanisms. (Non-pulmonary extrapolation). Direct experimental evidence within ARDS pulmonary immune-cell systems remains absent. The net biological readout depends on local metabolite availability within the pulmonary microenvironment. Pulmonary FXR/TGR5 receptor abundance shapes cell-specific vulnerability to bile-acid-modified ferroptosis; detailed intracellular signalling cascades are not recounted here. Clinical cohort-level associations link faecal secondary-bile-acid profiles remodelled by Bacteroides to respiratory-failure progression requiring mechanical ventilation, pointing toward a plausible gut-metabolite-driven contribution to ARDS-relevant lung injury.

#### 4.4.2 Cell-type-specific ferroptosis outcomes driven by bile acid–FXR/TGR5 interactions

In the ARDS lung, distinct pulmonary cell populations possess divergent bile-acid receptor expression profiles and thus respond variably to identical gut-derived bile-acid metabolite pools (112, 113). Consequently, the overall cellular ferroptosis threshold for each cell type is jointly determined by local bile-acid metabolite concentration together with cell-intrinsic FXR- versus-TGR5 receptor abundance. This cell-type-dependent susceptibility pattern awaits dedicated ARDS-specific experimental validation. Observations from non-pulmonary experimental systems provide only indirect mechanistic clues for potential pulmonary behaviour and cannot be directly extrapolated to lung pathology.

#### 4.4.3 Convergence with AhR/Nrf2 and TLR4/NF-κB networks

Bile-acid-responsive cellular states interface with the anti-ferroptotic AhR/Nrf2 and pro-ferroptotic TLR4/NF-κB-driven immunophenotypes (105, 114) to reshape macrophage profiles. Altered bile-acid-related conditions favour pro-inflammatory-dominated macrophage profiles and amplify LPS-provoked inflammatory responses (115, 116). In this manner, Bacteroides-derived bile-acid metabolites function as rheostats tuning pulmonary ferroptosis-resistance versus susceptibility (110, 112). The resulting phenotype remains cell-type-specific and governed by local receptor expression together with metabolite supply. Existing FXR/TGR5-bile-acid knowledge originates largely from metabolic- and hepatic-disease pre-clinical work; direct experimental evidence within ARDS pulmonary immune-cell systems remains absent. Findings originating from extra-pulmonary disease models are not expanded here.

### 4.5 Microbiota-host co-metabolism: bile acids and ferroptosis susceptibility

This section concentrates on gut-local microbial enzymatic remodelling of bile-acid pools rather than pulmonary signalling cascades. Potential ARDS-relevant pulmonary phenotypic outcomes inferred from indirect pre-clinical evidence for altered bile-acid profiles have already been described in Sections 4.3-4.4; therefore, full recapitulation of FXR/TGR5 intracellular signalling cascades is avoided here.Gut microbiota and the host jointly coordinate bile-acid metabolism, forming a central co-metabolic hub that shapes systemic ferroptosis susceptibility in ARDS.

#### 4.5.1 Microbial enzymatic conversion of primary to secondary bile acids

Bile acid transformation is a common gut microbiome activity that produces secondary bile acids, some of which are important for human health (117). One such process, 7α-dehydroxylation, converts the primary bile acids, cholic acid and chenodeoxycholic acid, to deoxycholic acid (DCA) and lithocholic acid (LCA), respectively (117). While the complex multi-step bile acid 7α-dehydroxylation pathway has been well-studied for conversion of “kinked” primary bile acids such as cholic acid (CA) and chenodeoxycholic acid (CDCA) to deoxycholic acid (DCA) and lithocholic acid (LCA), respectively, the enzymatic basis for the formation of “flat” stereoisomers (primary allo-bile acids) remains less explored (118). Bile acids deconjugation by bacterial bile salt hydrolase (BSH) has long been recognized as the first necessary bile acid modification required before further transformations can occur (119, 120). Bile salt hydrolase activity is common among human gut bacterial isolates spanning seven major phyla, though significant variation exists across taxa (119, 120). Notably, BSH possesses dual functions in bile acid metabolism: beyond deconjugation, it also acts as an amine N-acyltransferase to produce amino acid bile acid amidates that can affect host-mediated metabolic processes (121, 122).

#### 4.5.2 Taxa-specific bile acid remodeling and disease relevance

The expression of microbial bile salt hydrolase, bile acid operon (BaiCD) and hydroxysteroid dehydrogenases (hdhA) required for DCA and downstream metabolite synthesis provides a mechanistic basis for altered bile acid profiles with disease progression (123, 124). Bacteroidetes and several genera of the Lachnospiraceae family containing DCA-generating capacity are key contributors to this process (123, 124). Deoxycholic acid (DCA) and lithocholic acid (LCA) are secondary bile acid metabolites produced from primary bile acids by gut microbiota that play key immune regulatory functions by promoting FOXP3 regulatory T (Treg) cell differentiation (125). Even very low abundant bacteria can dramatically change the metabolic output: Clostridium scindens, for example, converts cholic acid into the secondary bile acid deoxycholic acid (110). Additionally, specific human gut bacteria and corresponding enzymes have been identified that convert the secondary bile acid lithocholic acid into 3-oxoLCA as well as the abundant gut metabolite isolithocholic acid (isoLCA) (112). BSH-encoding genera, particularly within the Lactobacillus genus, have been identified as crucial contributors, with 16 main species obtained using metagenomic data from publicly available human gut microbiome databases (125).

#### 4.5.3 Microbial bile acids and FXR/TGR5 modulation

Several microbe-derived bile acids attenuate farnesoid X receptor (FXR) activation, suggesting that loss of these metabolites during inflammation may increase FXR activity and exacerbate the course of disease (126). Bacteroides acidifaciens ameliorates liver injury through a bile salt hydrolase that generates unconjugated bile acids to activate intestinal FXR and its downstream target, fibroblast growth factor-15 (FGF15) (127). Conversely, downregulation of BSH activity by sedanolide increased the ratio of conjugated/unconjugated bile acids, thereby inhibiting the intestinal FXR pathway (126). A novel class of secondary bile acids with 3-O-acylation substitution inhibits the intestinal FXR; administration of C. minuta alleviated features of metabolic disease associated with a significant increase in these acylated bile acids, referred to as 3-O-acyl-cholic acids (128). Pulmonary ferroptosis-related phenotypic consequences arising from these gut-originated bile-acid shifts are described in Section 4.4 and are not recapitulated in this section.

#### 4.5.4 Bile acids, ferroptosis, and ARDS

This section focuses exclusively on gut-local microbial enzymatic remodelling of bile-acid pools. Pulmonary phenotypic outcomes potentially driven by these bile-acid modifications are elaborated in Section 4.4 and are not recapitulated here. Further ARDS-specific cell and animal studies are needed to dissect strain-specific microbial functions and design precision therapies targeting this core gut microbiota–ferroptosis metabolic cascade (summarized in Chapter 5). Analogous bile acid-ferroptosis regulatory patterns observed in metabolic and neoplastic diseases will not be elaborated here.

## 5 Therapeutic targeting of the microbiota–ferroptosis–immune loop

Based on the proposed gut microbiota–metabolite–ferroptosis–immune polarization loop discussed in Chapters 2–4, this chapter stratifies all potential intervention strategies along sequential pathological nodes: microbiota modulation as an early-stage therapeutic node, specific ferroptosis pharmacological inhibitors as mid-stream cytoprotective agents, and multi-omics biomarker-guided precision diagnosis as a downstream translational tool. All intervention strategies below are designed to interrupt different nodes along the complete pathological cascade: early-stage therapeutic node, ferroptosis inhibition midstream, and multiomics-based patient stratification downstream to break the macrophage ferroptosis-inflammation vicious cycle. **Figure 3** summarizes three major therapeutic directions targeting the microbiota–ferroptosis–immune loop: microbiota-based therapies, ferroptosis inhibitors, and multi-omics-driven precision medicine.

### 5.1 Ferroptosis inhibitors

I. Ferroptosis Inhibitors (Cytoprotective Agents).

Interventions described below are categorized according to available evidence: Human ARDS data; ALI/sepsis-ALI pre-clinical; Non-pulmonary data.

Key mechanistic pathways are further summarized with full evidence grading in **Table 3**.

**Table 3:** Evidence-grade summary of core mechanistic pathways linking the gut-lung axis, iron metabolism and ferroptosis in ARDS.

Pathway	Experimental model	Species/human population	Cell type	Intervention/exposure	Major finding	Evidence category
SCFA-mediated anti-ferroptosis gut-to-lung signalling	Sepsis-ALI animal model	Mouse/Rat	Alveolar macrophages, type-II alveolar epithelial cells	Faecal microbiota alteration/SCFA supplementation	SCFAs elevate cellular ferroptosis threshold and reduce pulmonary lipid peroxidation	Sepsis-ALI pre-clinical
LPS-TLR4/NF-κB pro-ferroptosis signalling	Sepsis-ALI animal; human ICU cohort	Mouse/Human	Alveolar macrophages, pulmonary endothelial cells	LPS challenge, gut dysbiosis induction	Translocated LPS drives pro-inflammatory, pro-ferroptotic macrophage activation	Sepsis-ALI pre-clinical; partial human ARDS data
NET-macrophage ferroptosis crosstalk	Hepatic IRI, non-pulmonary injury model	Mouse	Bone-marrow-derived macrophages, endothelial cells	NET induction/DNase-I NET-degradation	NETs trigger macrophage ferroptosis; effect in ARDS lung remains unvalidated	Non-pulmonary extrapolation
T-cell-driven ferroptosis in lung injury	Cancer/autoimmune disease models	Mouse	T lymphocytes	T-cell manipulation	T-cell ferroptosis contributes to tissue injury; no ARDS-specific validation	Non-pulmonary extrapolation
Bile-acid-FXR/TGR5 modulation of macrophage ferroptosis	Liver/metabolic disease pre-clinical	Mouse	Hepatic/immune cell lines	Bile-acid agonist/antagonist	Bile-acid-FXR/TGR5 axis modulates lipid peroxidation susceptibility; pulmonary ARDS data absent	Non-pulmonary extrapolation; Hypothetical
NCOA4-ferritinophagy-iron-driven ferroptosis in lung	LPS-induced ALI	Mouse	Alveolar macrophages, type-II alveolar epithelial cells	NCOA4 genetic modulation	NCOA4-dependent ferritinophagy releases labile iron promoting pulmonary ferroptosis	ARDS/ALI pre-clinical
Hepcidin regulation of iron homeostasis	LPS-ALI; human ARDS cohort	Mouse/Human	Whole-organism/serum biomarker	Hepcidin overexpression/neutralization	Hepcidin acts as an iron-homeostasis-regulating peptide; it modulates ferritin-dependent iron sequestration and alleviates LPS-ALI injury, but is not an upstream inducer of NCOA4-mediated ferritinophagy.	ARDS/ALI pre-clinical; partial human ARDS data

Evidence-category definitions: Human ARDS data, human ARDS-specific clinical or ex-vivo data; ARDS/ALI pre-clinical, pre-clinical ARDS/ALI experimental animals; Sepsis-ALI pre-clinical, sepsis-associated acute lung-injury animal studies; Non-pulmonary extrapolation, observations obtained from non-respiratory disease models; Hypothetical inference, mechanistic deduction without direct experimental support. Only core pathways are listed to avoid over-expansion.

Iron chelators: Deferoxamine (DFO) reduces labile iron pools, inhibits Fenton reaction-driven lipid peroxidation and alleviates ferroptosis in sepsis-induced acute lung injury preclinical models (ALI/sepsis-ALI pre-clinical) (129).

Lipid peroxidation scavengers: Ferrostatin-1 and liproxstatin-1 block phospholipid peroxidation and relieve LPS-triggered pulmonary ferroptosis (ALI/sepsis-ALI pre-clinical) (130–133).

GPX4/xCT pathway enhancers: Dipyridamole suppresses ferroptosis in pulmonary epithelial and endothelial cells by activating SOD1, which in turn inhibits the CREB1/HMOX1 pathway, thereby reducing lipid peroxidation (12). It alleviated lung damage in mouse ALI models and human alveolar organoids. A preliminary clinical trial has evaluated dipyridamole as adjuvant therapy for ARDS patients (ALI/sepsis-ALI pre-clinical; limited preliminary human ARDS data) (12). Note: The RNF126-SLC7A11-related mechanism is reported in non-pulmonary injury models and is not supported by reference 15; whether it functions in ARDS is still unknown.

Nrf2 activators: Sulforaphane activates the AMPK-Nrf2 axis to elevate ferritin and SLC7A11, inhibiting ferroptosis in sepsis-induced lung injury (ALI/sepsis-ALI pre-clinical) (134). ADSC-derived miR-125b-5p exosomes relieve PMVEC ferroptosis through the Keap1/Nrf2/GPX4 cascade and improve septic lung function (ALI/sepsis-ALI pre-clinical) (135).

Lipid-soluble antioxidants: Lipoprotein-bound vitamin E blocks lipid peroxidation to restrain ferroptosis in inflammatory lung injury(ALI/sepsis-ALI pre-clinical) (136).

Sepsis-induced ALI nanoparticle & biologic agents: CD8@PD-1-CTL-M@PLGA/Fer-1 nanoparticles carrying ferrostatin-1 reduce ferroptosis in sepsis-associated acute lung injury (ALI/sepsis-ALI pre-clinical) (137). YAP1 modulates multiple ferroptosis mediators to participate in septic ALI pathogenesis (ALI/sepsis-ALI pre-clinical) (138).

Other ARDS/ALI ferroptosis regulators: Metrnβ overexpression mitigates LPS-triggered lung inflammation and ferroptosis(ALI/sepsis-ALI pre-clinical) (139). FGF2 protects AT2 cells against ferroptosis in ALI models (ALI/sepsis-ALI pre-clinical) (140). Verteporfin improves ARDS outcomes by reshaping macrophage polarization (ALI/sepsis-ALI pre-clinical) (141). Electroacupuncture suppresses ferroptosis to alleviate LPS-induced ARDS (ALI/sepsis-ALI pre-clinical) (42).

II. Ferroptosis Inducers (Unsuitable for ARDS Lung Protection).

Evidence classification for microbiota-targeted therapies: Human ARDS data; ALI/sepsis-ALI pre-clinical; Non-pulmonary data.

xCT inhibitors deplete intracellular GSH to trigger ferroptosis and cannot be applied for pulmonary cytoprotection in ARDS. Erastin and sulfasalazine block cystine uptake to drive robust ferroptosis; they serve only laboratory tools and anti-tumor reagents and are excluded from ARDS therapeutic schemes.

Critical Clarifications.

Drug activity varies by cell type and disease background. This classification is based on protective phenotypes observed in ARDS and sepsis-related lung injury, rather than malignant disease models. Tanshinone is omitted due to insufficient lung-specific experimental evidence.

Some agents have shown efficacy in ARDS/sepsis-ALI models, but human ARDS-specific validation is lacking; many mechanistic data are extrapolated from non-pulmonary systems. Major translational barriers include poor lung bioavailability, undefined dosing standards and unconfirmed safety in critically ill ARDS patients (12).

Dedicated ARDS-specific clinical trials for ferroptosis-targeted pharmacological agents are still lacking (see Section 5.3 and Section 6 for detailed translational limitations).

### 5.2 Microbiota modulation

Evidence classification for microbiota-targeted therapies: Human ARDS data; ALI/sepsis-ALI pre-clinical; Non-pulmonary data.

Gut microbiota interventions regulate host ferroptosis susceptibility through microbial metabolites. Preclinical data indicate probiotics and high-fiber diets boost SCFA and polyamine synthesis to maintain redox balance and suppress lipid peroxidation; by contrast, dysbiotic metabolites such as hydrogen sulfide aggravate oxidative injury (11). While FMT and single-strain probiotics remodel bile acid pools and facilitate Treg differentiation to lower pro-inflammatory TNF-α/IL-6 in extra-pulmonary models, their protective effects vary greatly across experimental settings (142, 143). Such observations still require verification in ARDS-specific preclinical systems(ALI/sepsis-ALI pre-clinical; no dedicated human ARDS data).

Clinically, formulations including Lactobacillus rhamnosus GG and Bifidobacterium spp. are only tested on mixed ICU cohorts instead of strictly defined ARDS groups. General ICU randomized trials yielded inconsistent reductions in ventilator-associated pneumonia risk, alongside documented risks of probiotic-induced bacteremia (144). No Phase III trials enroll pure ARDS populations to evaluate ferroptosis-related clinical endpoints. FMT is well-established for recurrent Clostridioides difficile infection and inflammatory bowel disease (142, 143), yet no registered clinical studies investigate its gut-lung protective effects or ferroptosis-modulating capacity in ARDS (Non-pulmonary clinical experience; no ARDS-specific clinical trial data). Synbiotics combining Clostridium butyricum and pectin strengthen intestinal barrier and antioxidant function in preclinical assays, with no available human data for respiratory failure (145). Major unresolved gaps remain for ARDS translation: strain-specific activity, long-term intestinal colonization stability, standardized ICU dosage and subgroup stratification standards have not been clarified. Only tentative mechanistic deductions can be drawn from existing evidence. Well-designed ARDS randomized trials incorporating ferroptosis biomarkers and systematic safety monitoring are mandatory before clinical application; microbiota-targeted therapies for ARDS ferroptosis regulation remain largely experimental at present.

Robust ARDS-specific clinical evidence for microbiota-directed interventions remains absent (see Section 5.3 and Section 6 for detailed translational limitations).

### 5.3 Biomarker development and precision medicine

Biomarker development underpins personalized ARDS care. Multi-omics platforms integrate genomics, transcriptomics, proteomics and metabolomics to build comprehensive diagnostic panels. Gut microbiome signatures, together with ferroptosis-related metabolites (PUFAs, cysteine, MDA, 4-HNE), reflect pulmonary lipid peroxidation status. Immune biomarkers such as M1/M2 markers (iNOS, CD206), Treg/Th17 ratios and NET-related MPO/citrullinated histone H3 support ARDS subphenotyping and immunotherapy guidance (65, 146). Note: M1/M2 nomenclature originates largely from *in-vitro*/pre-clinical models and represents simplified biomarker labels for descriptive purposes only. A nested Phase 2a MSC trial cohort analyzed bronchoalveolar lavage biomarkers to decipher MSC-mediated lung injury relief, demonstrating multi-omics biomarker utility for tracking therapeutic responses (147). Machine learning algorithms combine microbiomic, metabolomic and transcriptomic datasets to stratify ARDS patients based on serum GPX4, ACSL4, iron indicators and gut flora profiles. Recent longitudinal multi-omics studies have identified reproducible molecular signatures associated with ARDS mortality, including redox impairment pathways that may intersect with ferroptosis biology (22). Multi-centre proteomic profiling has further demonstrated that ARDS subphenotypes exhibit distinct protein patterns with potential therapeutic implications (23). To date, however, no unified multi-omics biomarker panel incorporating gut microbiota and ferroptosis-related markers has been validated in human ARDS cohorts. Major translational barriers include inconsistent diagnostic cutoffs, wide inter-individual variation and the inability to perform real-time dynamic monitoring, with large multicenter cohort validation still lacking. Future research should prioritize AI-assisted diagnostic tools, cross-center verification and shared biomarker databases.

Collectively, the gut microbiota–ferroptosis axis presents a promising therapeutic framework for ARDS. Translational strategies converge on three pillars: (1) microbiota modulation (probiotics, prebiotics, FMT) restoring metabolic-immune homeostasis via SCFAs, indoles, and bile acids through the gut–liver–lung axis; (2) ferroptosis inhibition targeting iron metabolism, lipid peroxidation, and ferritinophagy; and (3) multi-omics-guided precision medicine enabling biomarker-informed patient stratification and therapy optimization. Ongoing clinical exploration — such as biomarker-embedded cell therapy trials — highlights the evolving bridge between mechanistic insights and bedside application.Biomarker screening targeting the gut microbiota-ferroptosis axis can support subphenotyping to realize targeted microbiota and anti-ferroptosis intervention for ARDS patients.

Although integrating multi-omics with biomarker panels offers theoretical foundations for stratifying acute respiratory distress syndrome (ARDS) subphenotypes (148, 149), numerous translational bottlenecks impede their widespread clinical implementation. Primarily, there is a conspicuous absence of standardized detection protocols; biomarkers from serum or bronchoalveolar lavage fluid—such as malondialdehyde, glutathione peroxid 4, and iron indicators—alongside gut microbiome data, lack universally accepted reference thresholds across institutions. Moreover, the substantial costs and prolonged turnaround times of these assays fail to satisfy the urgent diagnostic requirements of acute ARDS, where rapid identification of inflammatory subphenotypes is critical (27). Beyond static measurements, real-time dynamic monitoring remains unachievable. Given that gut microbial composition and pulmonary ferroptosis markers can fluctuate precipitously within hours as ARDS progresses, single time-point sampling inadequately captures the evolving disease activity, hindering the timely adjustment of targeted therapies (22). Finally, the field currently lacks validated stratification criteria. Without clinically established cutoff values to reliably segregate patients into high- versus low-ferroptosis subgroups, clinicians are unable to accurately identify candidates who would genuinely benefit from anti-ferroptosis agents or microbiota-modulating treatments (23). Consequently, bridging this translational gap necessitates rapid, standardized, and dynamically adaptable diagnostic frameworks to actualize precision medicine in ARDS (149, 150).

Despite encouraging preclinical efficacy, multiple translational hurdles limit clinical translation of ferroptosis inhibitors. Systemic administration leads to predominant hepatic and renal accumulation of small-molecule agents such as ferrostatin-1, liproxstatin-1 and dipyridamole; high systemic doses are required to reach therapeutic lung concentrations, causing hepatic lipid deposition and renal oxidative injury (12). Standardized inhalation formulations for ventilated patients remain unavailable, and targeted pulmonary delivery of dipyridamole still requires optimization (12).

In addition, a hypothesized therapeutic window exists from pre-clinical observations: ferroptosis-targeted agents may preferentially exert protective effects during early, reversible alveolar injury. However, this hypothesized therapeutic window has not been clinically validated in human ARDS cohorts. No rapid, non-invasive biomarkers exist to distinguish reversible early-stage ARDS from irreversible lung damage, leading to empirical, ineffective treatment in ICUs.

Safety risks are prominent for critical patients. Long-term intravenous deferoxamine may induce systemic iron deficiency and weaken innate immunity, raising secondary infection risk. Nrf2 agonists carry latent adverse effects under certain clinical contexts. Moreover, high-level clinical evidence remains limited to small animal and retrospective cohort data; no large Phase II/III randomized trials enrolling moderate-severe ARDS patients have been completed to validate unified dosing, infusion cycles and long-term safety profiles.

As of October 2026, no ferroptosis-targeted drug has gained official approval for ARDS treatment (12). Further ARDS-specific *in vitro* and *in vivo* experiments are needed to validate pulmonary delivery efficiency, *in vivo* efficacy and immunomodulatory functions of candidate agents. The pathogenic cascade of sepsis-associated ALI/ARDS centers on GPX4 inactivation, iron overload and lipid peroxidation, accompanied by endothelial and epithelial dysfunction, consistent with findings from preclinical and preliminary clinical research (46).

### 5.4 Similar practical translational obstacles also apply to microbiota-targeted therapeutic strategies for ARDS.

Despite the theoretical promise of modulating the gut microbiome to suppress ferroptosis-mediated inflammatory cascades (11, 27), several pragmatic and clinical limitations hinder the widespread adoption of such approaches in ARDS intensive care settings (11). The severely compromised intestinal mucosa in ARDS patients, marked by profound barrier disruption, impedes the stable engraftment of orally administered probiotics, while single-strain formulations fail to restore a balanced microbial ecosystem; although multi-strain synbiotics hold conceptual appeal, mature, ICU-compatible commercial products remain unavailable, and therapeutic outcomes fluctuate considerably depending on the degree of baseline dysbiosis (11, 151). Furthermore, a substantial subset of critically ill ARDS patients undergo deep sedation and mechanical ventilation, frequently complicated by paralytic ileus, gastrointestinal bleeding, or enteral feeding intolerance. In some critically-ill ARDS patients, oral probiotic supplementation may be practically challenging due to paralytic ileus, gastrointestinal bleeding or enteral feeding intolerance. FMT implementation faces substantial practical barriers including bowel preparation requirements and antibiotic withdrawal, which are often difficult to reconcile with time-critical acute ARDS management. These represent practical safety and feasibility concerns rather than formally proven absolute contraindications, and standardized enteral delivery regimens for synbiotics in the ICU context have yet to be formalized (13, 151).More critically, life-threatening safety concerns arise in immunocompromised hosts: live probiotic organisms such as Lactobacillus and Bifidobacterium species carry a documented, albeit rare, risk of inducing bacteremia or fungemia in ARDS patients with shock, impaired immunity, and a leaky gut barrier, with retrospective ICU cohort analyses having recorded fatal bloodstream infections following probiotic administration (152–156). Similarly, FMT introduces the potential for transmitting multidrug-resistant organisms, unidentified pathogens, and pro-inflammatory metabolites to vulnerable recipients (142, 143). Finally, the design of existing clinical trials exhibits notable shortcomings, as most randomized controlled studies enroll heterogeneous ICU populations rather than rigorously defined ARDS subgroups, prioritize ventilator-associated pneumonia incidence as the primary endpoint, and neglect ferroptosis biomarkers, alveolar injury indicators, or ARDS-specific mortality as core outcome measures; moreover, no stratified trials have been conducted based on baseline gut dysbiosis profiles, precluding the realization of precision microbiome-targeted interventions in this patient population (151).

In summary, while microbiota modulation and ferroptosis-targeted interventions hold substantial promise for disrupting the self-amplifying gut-lung inflammatory axis in acute respiratory distress syndrome (ARDS) (12, 157, 158), a multitude of unresolved translational bottlenecks severely impede their clinical implementation. Specifically, the clinical translation is constrained by a lack of ARDS-specific randomized trial evidence—highlighted by the current absence of FDA-approved therapeutics explicitly targeting ferroptosis for ARDS management (12)—alongside suboptimal lung-targeted drug delivery systems and ill-defined therapeutic windows. Furthermore, critically ill and mechanically ventilated patients face unique safety hazards, and the unpredictable heterogeneity of flora colonization complicates therapeutic efficacy, as evidenced by the distinct microbiome signatures characterized by the depletion of healthy core microbes and the enrichment of pathogenic strains like *Pseudomonas* and *Staphylococcus* in ARDS (151, 158). Overcoming these barriers would require standardized protocols, independent replication in diverse settings, and the integration of multi-target strategies beyond isolated ferroptosis modulation. These practical limitations are further elaborated as core research gaps in Section 6, which also outlines targeted future research directions aimed at surmounting these clinical obstacles and advancing precision microbiome and ferroptosis therapies for critically ill patients (151).

Because the complete Phase 1a–1b–2 (pulmonary-to-gut-to-pulmonary) feed-forward cascade (see **Box 1** for full definition of Phase 1a-1b-2 working model) has not yet been directly validated experimentally, the optimal timing of microbiota-modulating and anti-ferroptotic interventions remains uncertain. Future clinical trials should therefore consider stratifying patients by disease stage (e.g., early alveolar injury versus established ARDS) and intestinal barrier status when assessing therapeutic efficacy.

## 6 Challenges and future perspectives

Current research has multiple unresolved limitations centered on ARDS-specific evidence, which constitute our primary research priorities. First, large-scale human ARDS clinical cohorts and dedicated sepsis-induced ALI animal, primary alveolar cell studies remain scarce, and most mechanistic conclusions lack lung-specific verification. Second, strain-specific gut microbiota interventions and ferroptosis-targeted agents have not been validated in stratified ARDS randomized controlled trials, with undefined dosing, delivery routes and safety standards for mechanically ventilated ICU patients. Third, standardized multi-omics cut-off values for high/low ferroptosis ARDS subphenotypes are absent, restricting precision patient stratification. By contrast, regulatory interactions between tumor, hepatic IRI and metabolic disease models only serve as secondary indirect references and will not be elaborated here. Future research should first prioritize ARDS-oriented *in vitro*, *in vivo* and clinical validation, followed by exploratory cross-disease mechanistic comparison if required.

Four major interconnected knowledge gaps persist in this research field: (i)shortage of temporally resolved experimental evidence supporting the pulmonary-to-gut limb of the hypothetical Phase 1a–1b–2 cascade(see **Box 1** for full definition of Phase 1a-1b-2 working model); (ii) observational human gut-microbiota association studies require proper adjustment for well-known ICU-related confounders (see Section 3.1.1); (iii) incomplete mechanistic dissection of how isolated ARDS pulmonary injury disturbs intestinal-barrier integrity; (iv) scarcity of delayed-intervention pre-clinical studies verifying whether gut-targeted manipulation can reverse already-established pulmonary injury after ARDS onset. Priority should be given to temporally separated ARDS animal models and adequately adjusted human cohort analyses.

Specifically, strain-specific microbial regulatory functions are poorly characterized; existing work only describes global gut dysbiosis without strain-level functional verification, restricting precision microbiota therapy for ARDS. Further, large human ARDS multi-omics cohort data are lacking, and unified analytical pipelines for patient subtyping have not been established. In addition, high-quality ARDS-specific clinical trials of ferroptosis inhibitors, probiotics and FMT are absent; standardized dosage, administration routes and ICU safety standards remain undefined. Finally, the spatiotemporal dynamics of the gut-lung microbiota-ferroptosis loop across ARDS disease stages and distinct pulmonary cell populations remain underexplored. Crosstalk between ferroptosis, pyroptosis, necroptosis also lacks systematic ARDS-oriented validation. Corresponding mechanistic observations from tumor, hepatic ischemia-reperfusion and metabolic disease models are only secondary auxiliary references and will not be further discussed.

Future research should prioritize ARDS-specific experimental and clinical validation: spatial multiomics, lung organoids and single-cell sequencing can dissect cell-type and stage-specific changes of the gut microbiota-ferroptosis axis in sepsis-induced ALI and human ARDS samples. Research priorities include dynamic gut-microbe crosstalk tracking, strain-specific precision microbial interventions, and multi-omics diagnostic pipelines for ferroptosis subphenotyping. Human ARDS cohort validation is the most urgent unresolved translational gap. Comparisons of microbial-ferroptosis signaling across tumor, liver and metabolic diseases can be carried out as secondary exploratory research if necessary. For detailed discussion of experimental validation strategies for this cascade, see Chapter 3, Section 3.4.

From a translational standpoint, multiple bottlenecks block the clinical application of microbiota and ferroptosis modulators in ARDS. Top priorities include developing low-toxic inhaled lung-targeted ferroptosis inhibitors, ICU-adapted multi-strain synbiotics with standardized enteral delivery, and large stratified ARDS randomized trials using ferroptosis biomarkers as primary endpoints. Low-cost rapid multi-omics testing platforms are also required for real-time patient stratification. Related findings from extra-pulmonary tumor and hepatic injury models only serve as minor supplementary references and receive no in-depth discussion in this work.

## 7 Conclusions

This review proposes a conceptual bidirectional framework centered on the gut–lung axis: ARDS-associated pulmonary inflammation may contribute to intestinal barrier and microbial homeostasis disruption, reshaping the spectrum of circulating gut metabolites. These systemic microbial signals may remodel pulmonary macrophage iron and lipid metabolism, shift macrophage functional states, and promote ferroptosis in alveolar and endothelial cells, ultimately forming a self-reinforcing inflammatory amplification loop. Metabolites secreted by commensal flora may control cellular ferroptosis through direct and indirect signaling pathways. Herein, we construct a testable conceptual gut microbiota-ferroptosis immunometabolic circuit and bidirectional pathological loop, laying a structured theoretical foundation for ARDS patient subphenotyping and candidate targeted intervention.

Key challenges include defining strain- and metabolite-specific regulation of ferroptosis, conducting ARDS-specific clinical trials for probiotics and ferroptosis inhibitors, and developing standardized multi-omics and AI-assisted diagnostic tools. In summary, the gut microbiota-ferroptosis axis proposed in this work may deliver a novel mechanistic perspective for ARDS pathogenesis. The microbiota-ferroptosis interaction should be regarded as a testable research framework that may generate ARDS-specific mechanistic and translational hypotheses, rather than representing already-validated diagnostic biomarkers or stratified therapeutic targets for clinical application.
